# Comparative evolution of vegetative branching in sorghum

**DOI:** 10.1371/journal.pone.0255922

**Published:** 2021-08-13

**Authors:** WenQian Kong, Pheonah Nabukalu, T. Stan Cox, Valorie Goff, Jon S. Robertson, Gary Pierce, Cornelia Lemke, Rosana Compton, Jaxk Reeves, Andrew H. Paterson

**Affiliations:** 1 Plant Genome Mapping Laboratory, University of Georgia, Athens, Georgia, United States of America; 2 Department of Statistics, University of Georgia, Athens, Georgia, United States of America; 3 The Land Institute, Salina, Kansas, United States of America; National Institute for Plant Genome Research, INDIA

## Abstract

Tillering and secondary branching are two plastic traits with high agronomic importance, especially in terms of the ability of plants to adapt to changing environments. We describe a quantitative trait analysis of tillering and secondary branching in two novel BC_1_F_2_ populations totaling 246 genotypes derived from backcrossing two *Sorghum bicolor* x *S*. *halepense* F_1_ plants to a tetraploidized *S*. *bicolor*. A two-year, two-environment phenotypic evaluation in Bogart, GA and Salina, KS permitted us to identify major effect and environment specific QTLs. Significant correlation between tillering and secondary branching followed by discovery of overlapping sets of QTLs continue to support the developmental relationship between these two organs and suggest the possibility of pleiotropy. Comparisons with two other populations sharing *S*. *bicolor* BTx623 as a common parent but sampling the breadth of the Sorghum genus, increase confidence in QTL detected for these two plastic traits and provide insight into the evolution of morphological diversity in the Eusorghum clade. Correspondence between flowering time and vegetative branching supports other evidence in suggesting a pleiotropic effect of flowering genes. We propose a model to predict biomass weight from plant architecture related traits, quantifying contribution of each trait to biomass and providing guidance for future breeding experiments.

## Introduction

Plant architecture is the three-dimensional organization of a plant body. The above-ground architecture includes the pattern of vegetative branching, sizes and shapes of stalks, leaves and floral organs, and plant height. The expression of plant architecture varies during different developmental stages by a series of highly regulated endogenous genetic factors [1–3] and exogenous constraints exerted by environments. Genetic factors impart the biodiversity of plant architecture, contributing to adaptation to different niches, are often utilized in the classification of taxa. On the other hand, responsiveness to biotic and abiotic stresses tailors plant architecture to fitness under different environments [4,5].

Important aspects of plant architecture are tillering and vegetative branching, which are considered to be medium to low heritability traits with a high degree of plasticity [6,7]. The complexity of these traits is due in part to their non-deterministic and genetic pathways controlling axillary meristem initiation and outgrowth that affect the number of tillers and patterns of vegetative branching [2,3,8]. Many of these genes are involved in the production, signal transduction, transport, degradation and interactions of hormones such as auxin, cytokinin and strigolactone. Those hormones act directly and locally to promote or repress axillary meristem activity [7,9–12].

Recent studies have also suggested that genes involved in controlling flowering time also influence the activity of axillary meristems and thus influence tillering and vegetative branching. For example, the flowering locus T (*Ft*) gene that regulates flowering time in many species, has recently been found to trigger storage organ formation through direct interaction with the TCP factors [13]. The rice homolog of *Leafy* (*Lfy*) from Arabidopsis, expressed during the development of axillary bud and inflorescence branch primordia, is also required to produce tillers and panicle branches (Rao, 2008) [37].

As a C4 model plant, sorghum has a relatively small genome (~730 Mb) with a high quality reference genome sequence [14] and provides many avenues to study traits related to plant architecture. Using colinearity, results from sorghum may be extrapolated to many other C4 plants with large genomes, such as sugarcane. The flexibility to make crosses between the five main sorghum races (bicolor, guinea, caudatum, durra and kafir), and with wild relatives such as *S*. *propinquum* and *S*. *halepense* which vary widely in plant architecture, makes sorghum particularly attractive to dissect and compare genetic components of plant architecture. Compared to voluminous studies of plant height and flowering [15–24], understanding of genetic components for tiller number and vegetative branching in sorghum has been relatively limited [6,25–28], possibly due to difficulties in phenotyping and the lower heritability of these traits.

Here, we describe a quantitative trait study of two important components of plant architecture, tillering and vegetative branching, in two half-sib tetraploid BC_1_F_2_ populations derived from crossing *Sorghum bicolor* BTx623 and *Sorghum halepense* Gypsum 9E. A two-year, two-environment phenotypic evaluation in Bogart, GA and Salina, KS permitted us to identify major effect and environment specific QTLs [29,30]. Quantitative trait loci (QTLs) discovered in these two populations are compared to those from two diploid sorghum recombinant inbred line (RIL) populations sharing BTx623 as a common parent but sampling the breadth of the Sorghum genus, one a cross to *S*. *bicolor* IS3620C [31], and the other to *S*. *propinquum* [32]. QTLs identified in this study and their comparison elucidate morphological evolution in the Sorghum genus, are of practical use for marker-assisted breeding, and provide a foundation for molecular cloning and functional analysis.

## Materials and methods

**Fig 1:**
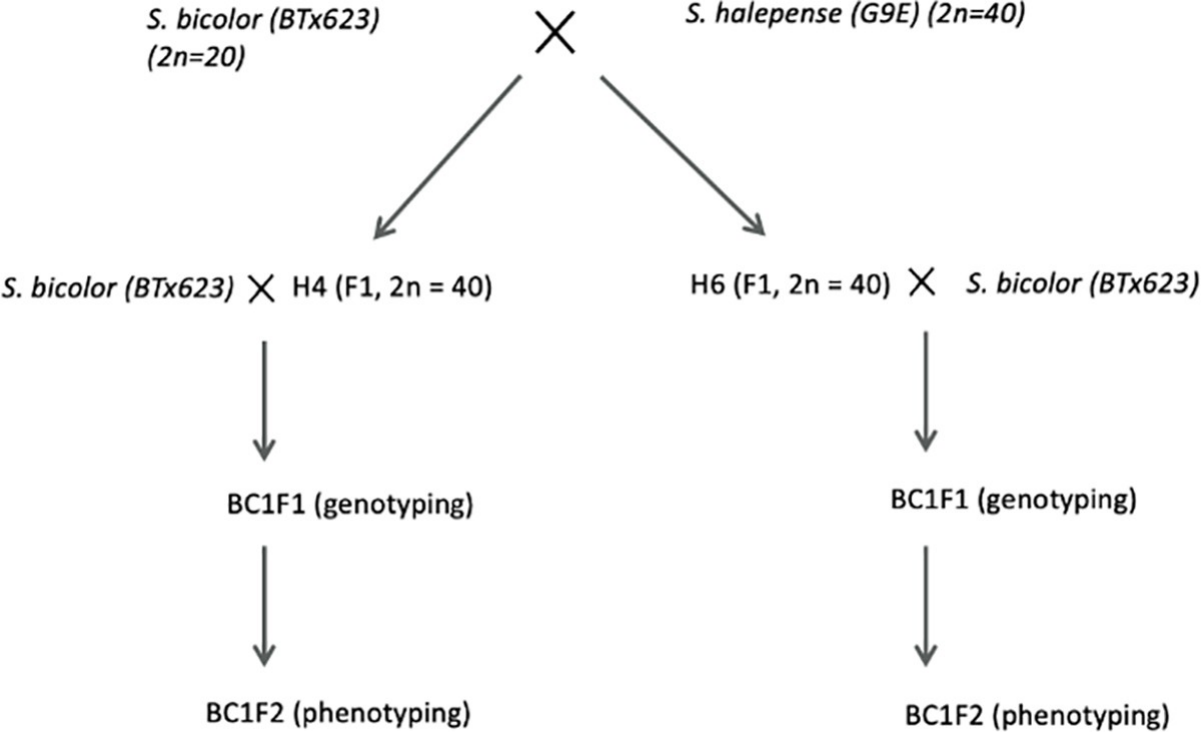
Population development of the two BC1F2.

Population development is shown in Fig 1. Genetic maps of two BC1F1 populations derived from crosses of *S*. *bicolor* (sorghum) and *S*. *halepense* were produced with totals of 722 and 795 single nucleotide polymorphism (SNP) markers. These maps respectively span 37 and 35 linkage groups, with 2–6 for each of the 10 basic sorghum chromosomes due to fragments covering different chromosomal portions or independent segregation from different *S*. *halepense* homologs. Details of population development, genotyping methods and methods for QTL analysis were discussed in Kong, Nabukalu [29] and [30].

### Phenotyping

We evaluated tillering (**TL**) and secondary branching per tiller (**BRCH**) in the BC_1_F_2_ families with three subsamples for each genotype in two fields in two years, 2013 and 2014; and at two locations, on May 29^th^ 2013 and Jun 9^th^ 2014 at the University of Georgia Plant Science Farm, Watkinsville, GA, USA (33.87°, -83.53°, Athens 2013 and Athens 2014 hereafter), and on Jun 3^rd^ 2013, and Jun 17^th^ 2014 at The Land Institute, Salina, KS, USA (38.77°, -97.57°, Salina 2013 and Salina 2014 hereafter). Tillering (**TL**) was measured by counting the number of tillers with mature seed heads before plants were senesced. Secondary branches per tiller (**BRCH**) was calculated by taking the average number of secondary branches from two representative tillers (S1 and S2 Files).

Phenotyping of vegetative branching in the IS-RIL population was consistent with our system applied to the *S*. *bicolor* × *S*. *propinquum* RILs described in Kong, Guo [6]. To compare secondary branching across population and environments, we used the number of mature tillers (**TL**), and calculated the average number of secondary branches per mature tiller (**BRCH**) in the IS-RIL and PQ-RIL population. The variance component method was used to calculate broad-sense heritability [H = V_G_/(V_G_+V_GE_/e +V_residual_/er)] where V_G_ is the variance estimate for genotype, V_E_ is the variance estimate for environment, V_GE_ is the genotype by environment interactions, e is the number of environments and r is the number of subsamples.

### Genetic analysis

To fully utilize the available data while protecting against false-positive results, genetic analysis employed two approaches. Using genetic maps that were constructed as described [29] from selected well-groomed SNP segregation data for each of the two SBSH-BC_1_F_2_ populations, interval mapping was conducted [33]. Permutation tests (with α = 0.10) suggested LOD scores of 2.9 and 3.1 for H4 and H6 populations, respectively. QTLs with LOD scores of 2.5 were listed as marginal QTLs. Additional QTLs were added to the model after considering the larger effect QTL as a fixed effect. For each trait, percentage of variance explained were calculated based on an additive QTL model with QTL positions refined.

In addition, single marker analysis was conducted using each SNP marker that met quality standards described (whether in the genetic map or not), using hierarchical clustering to separate SNP markers on potentially different homologous chromosomes and inferring QTLs only if more than 4 SNPs were found within a cluster cut at height of 0.3 in recombination frequency to mitigate spurious associations [30]. Similarities and differences in the results of these analyses were addressed in results.

### Mixed modeling for biomass

We constructed a mixed effect model with **Biomass** as the response variable; **FL**, **PH**, **TL**, **BRCH**, mid-stalk diameter (**MD**), the number of nodes (**ND**), and population (H4 or H6) as fixed explanatory variables; and the environment (**ENV**) as a random effect. **MD** was the stalk diameter at the middle of a plant. The average of the six phenotypes from two blocks and three subsamples was taken for the mixed effect modeling. A natural log transformation was used for **Biomass** to normalize the data. Mixed effect modeling and model selection used the lme4 and lmerTest packages in R [34,35]. We used a modified method to calculate R-squared for the fixed and model effects [36] for the mixed effect modeling.

## Results

### Summary statistics and heritability analysis

The average number of mature tillers (**ML**) of *S*. *halepense* G9E was 16, higher than the 2.6 of diploid *S*. *bicolor* BTx623 (S1 Table). Tetraploid BTx623 had an average of 0.77 more tillers than diploid BTx623 in 2013 (t = 2.91, p = 0.006) and 1.58 more in 2014 (t = 3.82, p = 0.0005). In the BC_1_F_2_ population, average **TL** for most lines fell within the range of those of their parents, showing less transgressive segregation than plant height (**PH**) and flowering time (**FL**) [30]. The average **TL** was 1.46 more in Salina than Athens (t = 14.07, p<0.001). Average **TL** in Athens was 2.24 (t = -21.87, p<0.001) fewer in 2013 than 2014; and in Salina 2013 was 2.14 fewer in 2013 than 2014 (t = 19.07, p<0.001). Average **TL** of the BC_1_F_2_ population is 0.30 greater than that of the PQ-RILs (t = 2.52, p = 0.020, S2 Table), and 2.83 greater than that of the IS-RILs (t = 36.19, p<0.001, S3 Table). Broad sense heritability estimates for **TL** were intermediate for all three populations, at 35%, 36%, and 30% for the PQRIL, ISRIL and SH-BC_1_F_2_ populations, respectively (S1–S3 Tables).

The number of secondary branches per primary tiller (**BRCH**) is sensitive to environmental changes and is also a fail-safe for a plant in case the primary seed head is damaged. Average **BRCH** of *S*. *halepense* is 13, dramatically higher than the 0.286 of *S*. *bicolor* BTx623 (S1 Table). There were no statistically significant differences for **BRCH** between diploid and tetraploid BTx623 in Athens 2014, Salina 2013 and 2014, while there was 2.1 more **BRCH** in tetraploid BTx623 than in diploid BTx623 (t = 4.16, p = 0.0011) in Athens 2013. The average number of **BRCH** of most progeny lines fell within the range of the respective parents. For the SH-BC_1_F_2_ progeny lines, the average number of **BRCH** in Athens was 1.29 more than in Salina (t = 25.50, p<0.001). Average **BRCH** in Athens was 0.45 more in 2013 than 2014 (t = 7.70, p<0.001); and in Salina was 0.60 more in 2013 than 2014 (t = 7.98, p<0.001). The average number of **BRCH** of the SH-BC_1_F_2_ population was 2.28 smaller than that of the PQ-RIL population (t = -14.38, p<0.001, S2 Table), and 0.99 smaller than that of the ISRIL population (t = -0.99, p<0.001, S3 Table). Broad-sense heritability estimates for **BRCH** are relatively low, 7% and 10% for the PQ-RIL and SH-BC_1_F_2_ populations, respectively, but intermediate for the ISRIL population, 40.9% (S1–S3 Tables).

### Trait correlations

**Fig 2:**
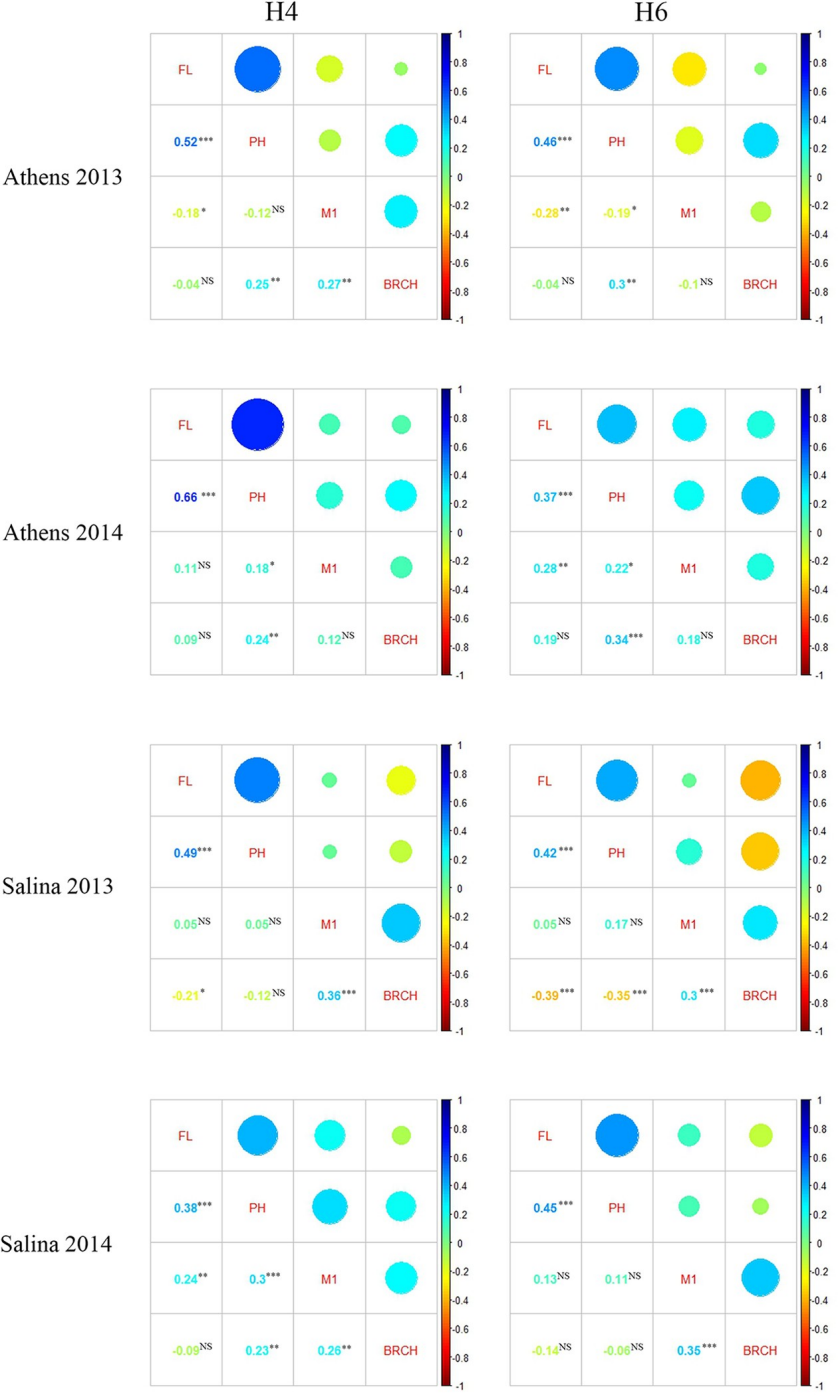
Correlation coefficients among days to flowering (FL), plant height (PH), number of mature tillers (TL) and number of secondary branches (BRCH) in the H4 and H6- derived SBSH-BC_1_F_2_ populations in four environments.

In all four environments, **FL** is positively and significantly correlated with **PH** (Fig 2) [30], i.e., late flowering individuals are generally taller than early flowering ones. **FL** and **TL** are negatively correlated in both the H4 (p = 0.034) and H6- derived populations (p = 0.032) in Athens in 2013, and positive in the other three environments, although not significant (p>0.05) for H4-derived populations in Athens 2014 or Salina 2013 and the H6-derived population in Salina 2014. In three out of four environments, Athens 2013, Salina 2013 and 2014, **FL** and **BRCH** are negatively correlated, with a non-significant positive correlation in Athens 2014. Correlations between **TL** and **BRCH** are generally positive, except for the H6 population in Athens 2013 where the correlation is negative but not significant.

### Genetic analysis

#### Number of tillers

We detected a total of two marginal QTLs, qTL.4A.H4.1 and qTL.4D.H4.1, for **TL** in the H4-derived population (Table 1). qTL.4A.H4.1 is significant in both Athens 2013 and Salina 2014, and qTL.4D.H4.1 is significant in Salina 2013 and Salina 2014. An additive model of the two QTLs, qTL.4A.H4.1 and qTL.4D.H4.1 explains 13.9% of the total phenotypic variance in Salina 2014. Although the peaks of qTL.4A.H4.1 are ~26 cM apart in Athens 2013 and Salina 2014, their corresponding physical locations of one-lod interval in genetic distance overlap. No QTLs for **TL** were detected in Athens 2014. Both QTLs have positive allele effects, indicating that *S*. *halepense* alleles increase **TL**.

**Table 1:** Parameters of TL (mature tillers) QTLs from interval mapping of the H4 and H6 SBSH-BC_1_F_2_ populations.

QTL Name	Pos (CM)	Pos (Mb)	LOD	% of Variance explained	Effect	Left (Mb)	Right (Mb)	Env
qTL.4A.H4.1	123.2	53.4	2.6	7.94	0.58	52.5	61.2	AT13
qTL.4A.H4.1	149.0	58.9	2.6	8.53	1.01	57.4	61.2	SL14
qTL.4D.H4.1	28.0	61.8–62.5	**3.2**	9.54	0.87	53.1	65.8	SL13
qTL.4D.H4.1	29.4	61.8–62.5	2.5	8.21	1.03	53.1	65.8	SL14
qTL.1D.H6.1	100.4	65.3	2.8	11.42	1.38	19.2	65.3	AT14
qTL.2C.H6.1	108.0	9.0	3.0	8.34	1.21	8.4	65.8	AT13
qTL.2C.H6.1	110.0	9.0	2.5	10.68	1.19	8.4	65.8	SL13
qTL.3E.H6.1	205.0	59.7	2.9	11.15	1.05	4.5	59.7	SL13
qTL.6A.H6.1	186.0	57.5	3.0	11.81	0.73	56.5	60.5	AT13
qTL.6B.H6.1	121.0	47.2	**6.7**	24.58	-1.09	47.2	50.9	AT13
qTL.9B.H6.1	55.0	53.6	**3.2**	12.57	0.78	47.9	55.8	AT13
qTL.10C.H6.1	87.7	6.0	2.8	11.69	-0.74	1.2	12.8	AT13

LOD scores in bold suggest significance beyond the permutation test.

We detected a total of two QTLs and five marginal QTLs for the number of **TL** in the H6-derived population with only qTL.2C.H6.1 significant in both Athens 2013 and Salina 2013 (Table 1). Five QTLs detected in Athens 2013, qTL.2C.H6.1, qTL.6A.H6.1, qTL.6B.H6.1, qTL.9B.H6.1 and qTL.10C.H6.1, collectively explain 34% of the total phenotypic variance, one QTL detected in Athens 2014 explains 11.42% of the total phenotypic variance, and two QTLs detected in Salina 2013 explain 13.9% of the total phenotypic variance. No QTLs were found in Salina 2014. While most QTL alleles have positive allele effects, indicating that *S*. *halepense* alleles increase **TL,** two marginal loci (qTL.6B.H6.1 and qTL.10C.H6.1) detected in Athens 2013 have negative allele effects, with *S*. *halepense* alleles decreasing the number of tillers.

**Fig 3:**
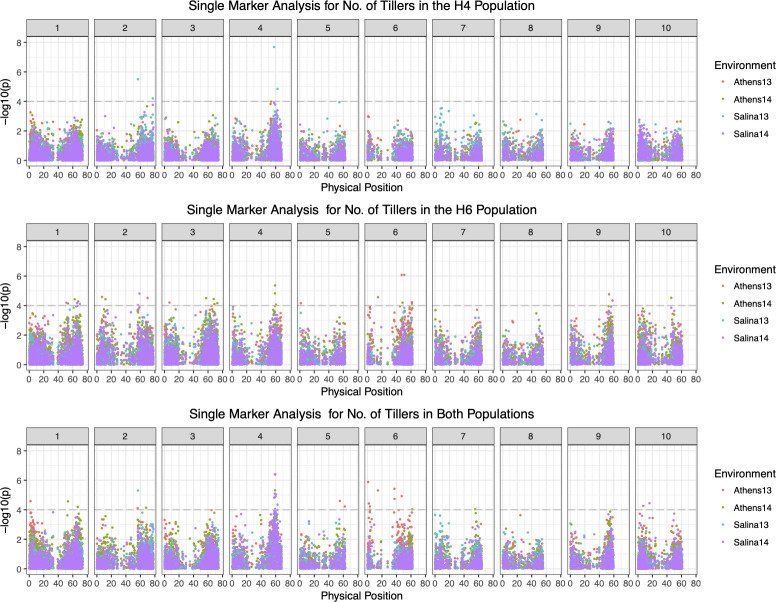
Single marker analysis of the number of mature tillers in the H4, H6 and pooled SBSH BC_1_F_2_ population.

Using single marker analysis, we detected a total of 63, 46, 26 and 48 significant SNP markers (p<10^−3^) for **TL** for pooled (H4 and H6) data in Athens 2013, Athens 2014, Salina 2013 and Salina 2014, respectively, with only one SNP marker, S4_58879601, significant in all environments (S1 Fig and Fig 3). Fewer signals detected for **TL** in multiple environments reflect lower heritability and large genotype by environment interactions. In the H4 population, we detected two QTLs for **TL**, qTL2.H4.1 and qTL.H7.1 in addition to the two QTLs on chromosome 4 detected by interval mapping (S4 Table). As was true for H4 QTLs found by interval mapping, *S*. *halepense* alleles increase the number of **TL**. In the H6 population, we detected a total of 14 QTLs for **TL** on chromosomes 1 (2), 2, 3(2), 4 (2), 6 (3), 9 (2), 10 (2) with three new QTLs (qTL4.H6.1, qTL4.H6.2 and qTL10.H6.2) not overlapping with any QTLs detected in the interval mapping, all with *S*. *halepense* alleles increasing the number of **TL** (S4 Table). The other 11 QTLs from the single-marker analysis overlap with seven QTLs from interval mapping based on their physical positions. We consider all marginal **TL** QTLs from interval mapping to be real QTLs, since results from single-marker analysis suggested lower P- values (smaller than 0.0001) for the peak SNPs (S4 Table).

#### Number of secondary branches per primary branch (BRCH)

We detected a total of five QTLs and two marginal QTLs for **BRCH** in the H4-derived population, including six from Athens 2014 and one from Salina 2013 (Table 2). No QTLs were found in Athens 2013 or Salina 2014. The six QTLs detected in Athens 2014 together explain 22.0% of the total phenotypic variance, while the one QTL detected from Salina 2013 explains about 8.28% of the phenotypic variance. It is interesting that six out of seven QTLs show negative allele effects (Table 2), suggesting that *S*. *halepense* alleles contribute to decreased **BRCH**, which is unexpected and contrary to the difference between parents. Those QTLs with negative additive effect might reflect late release of apical dominance from *S*. *halepense*, which is associated with fewer **BRCH**.

**Table 2:** Parameters of branching (BRCH) QTLs from interval mapping of the H4 and H6 SBSH-BC_1_F_2_ populations.

QTL Name	Pos (cM)	Pos (Mb)	LOD	%Var Explained	Effect	Left (Mb)	Right (Mb)	Env
qBRCH.1F.H4.1	4.0	1.6–3.2	**3.7**	11.56	-0.47	1.6	8.6	AT14
qBRCH.2D.H4.1	122.0	74.5	**3.0**	9.98	-0.38	66.1	75.5	AT14
qBRCH.4C.H4.1	8.0	4.8	2.6	8.48	-0.34	3.7	6.9	AT14
qBRCH.4D.H4.1	102.7	61.2	**3.2**	10.22	-0.38	20.7	61.8	AT14
qBRCH.5C.H4.1	59.8	11.6	**3.6**	11.46	-0.38	1.7	57.9	AT14
qBRCH.6B.H4.1	8.2	0.9	2.6	8.28	-0.48	0.9	37.2	SL13
qBRCH.7C.H4.1	86.0	61.6	**3.1**	9.96	0.36	56.5	62.8	AT14
qBRCH.1C.H6.1	142.0	70.2	**3.5**	13.82	0.41	69.1	72.5	AT14
qBRCH.3E.H6.1	203.0	59.7	**3.8**	13.72	0.95	4.5	59.7	SL13
qBRCH.3E.H6.1	218.0	59.7	**3.9**	7.91	0.88	2.7	59.7	SL14
qBRCH.5C.H6.1	6.0	54.5	**6.1**	19.19	1.37	2.6	3.1	AT13
qBRCH.6B.H6.1	20.0	3.1	**3.5**	13.72	-0.63	2.0	42.2	SL13
qBRCH.6B.H6.2	95.0	47.0	2.9	11.66	-0.65	3.3	50.9	SL14
qBRCH.10C.H6.1	19.2	2.4	**4.1**	16.55	1.11	2.4	58.3	SL13
qBRCH.10C.H6.2	91.0	6.0	2.8	11.76	-0.38	1.2	53.4	AT14

LOD scores in bold suggest significance beyond the permutation test.

We detected a total of five QTLs and two marginal QTLs for **BRCH** in the H6-derived population, with one QTL, qBRCH.3E.H6.1, significant in two environments, Salina 2013 and 2014 (Table 2). Two **BRCH** QTLs found in Athens 2014, qBRCH.1C.H6.1 and qBRCH.10C.H6.2, three BRCH QTLs found in Salina 2013, qBRCH3E.H6.1, qBRCH6B.H6.1,qBRCH10C.H6.1, and two BRCH QTLs found in Salina 2014, collectively explain 19.3%, 19.5% and 26.4% of the total phenotypic variance, respectively. For four QTLs, qBRCH.1C.H6.1, qBRCH.3E.H6.1, qBRCH.5C.H6.1 and qRBCH10C.H6.1, *S*. *halepense* alleles increase **BRCH** as predicted based on the parental phenotypes, while *S*. *halepense* alleles decrease **BRCH** for the other three QTLs, qBRCH6b.H6.1, qBRCH.6B.H6.2 and qBRCH.10C.H6.2.

**Fig 4:**
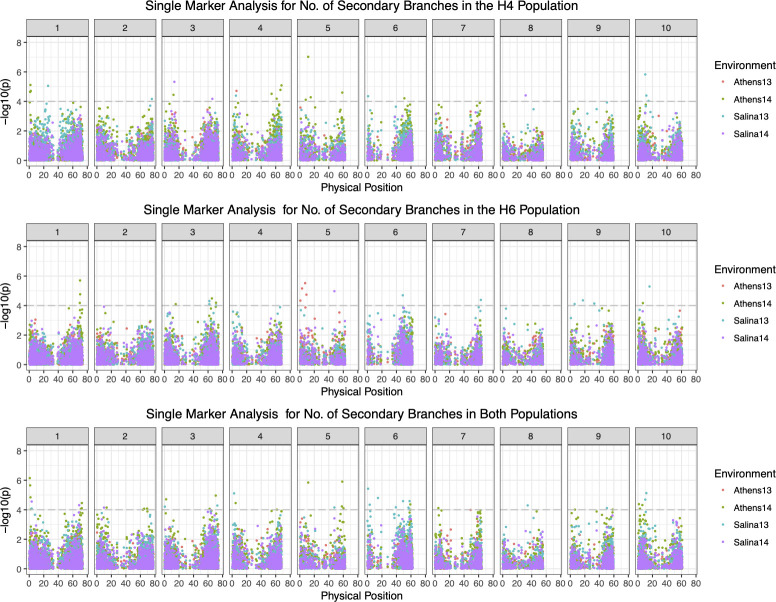
Single marker analysis for the number of secondary branches per tiller in the H4, H6-derived and the pooled BC_1_F_2_ populations.

We detected a total of 4, 110, 65 and 20 significant SNP markers (p<10^−3^) for **BRCH** in Athens 2013, Athens 2014, Salina 2013 and Salina 2014 for pooled data with very little correspondence among different environments. This observation is consistent with low heritability estimates and large genotype by environment interactions (S2 Fig and Fig 4). In the H4-derived population, we detected a total of 11 QTLs for **BRCH** on chromosomes 1 (2), 3, 4 (2), 5, 6(2), 7, 9, 10, with three negative effect QTLs, suggesting that *S*. *halepense* alleles at these loci decrease **BRCH** (S5 Table). A total of four QTLs, qBRCH1.H4.2, qBRCH3.H4.1, qRBCH9.H4.1 and qBRCH10.H4.1 were newly detected only in the single-marker analysis, all with *S*. *halepense* alleles increasing **BRCH**. In the H6 population, we detected a total of 11 QTLs on chromosomes 1, 3 (2), 4, 5, 6(3), 7, 9 10 with only one negative effect QTL, qBRCH.H6.2 (Table 2). A total of three QTLs, qBRCH4.H6.1, qBRCH7.H6.1 qBRCH9.H6.1, were newly detected in the single-marker analysis, with all three increasing **BRCH**. The other 8 QTLs detected in single markers analysis overlap with the seven BRCH QTLs from interval mapping by comparing their physical positions. We consider all marginal **BRCH** QTLs from interval mapping to be the real QTL, since single-marker analysis suggested lower P-values (smaller than 0.0001) for the peak SNPs (S5 Table).

### QTL correspondence across traits in the BC_1_F_2_ population

In most environments, **TL** and **BRCH** are significantly and positively correlated (Fig 2), therefore some QTL regions are expected to overlap due to their developmental relationship [6]. Indeed, we found two **TL** QTLs, qTL2.H4.1 and qTL.4D.H4.1 overlapping with qBRCH.2D.H4.1 and qBRCH.4D.H4.1 in the H4-derived population based on their physical positions. Four QTLs, qTL.3E.H6.1, qTL.6B.H6.1, qTL6A.H6.1 and qTL.10C.H6.1 overlap with qBRCH.3E.H6.1, qBRCH.6B.H6.2, qBRCH6.H6.3 and qBRCH.10C.H6.2 in the H6-derived population, respectively. Interestingly, *S*. *halepense* contributed opposite allele effects for the two pairs of overlapping QTLs in the H4-derived population (*S*.*halepense* alleles increased **TL** but decreased **BRCH**), but the same effect for all overlapping pairs in the H6-derived population.

Recent studies have suggested that genes controlling days to flowering might also influence tillering and vegetative branching [1,13,37,38]. We found a total of six **TL** QTLs overlapping with **FL** QTLs in the H6-derived population, with two pairs of QTLs, qTL.4.H6.1 with qFL4A.H6.1 and qTL6B.H6.1 with qFL6B.H6.2, showing opposite effects from *S*. *halepense* (Table 3).

**Table 3:** Comparisons of TL and FL QTL in the SBSH-BC_1_F_2_, IS-RIL and PQ-RIL population.

QTL Name	ISRIL	PQRIL	FLQTL
qTL2.H4.1 (+)		qM1_2.1 (+)	
qTL.4A.H4.1 (+)		qTL4.1 (-)^1^	
qTL.4D.H4.1 (+)		qTL4.1 (-)^1^	
qTL7.H4.1 (+)	qTL7.1 (+)	qTL7.1 (+)	
qTL.1D.H6.1 (+)	qTL_1.1 (+)		qFL1C.H6.1 (+)
qTL.2C.H6.1 (+)		qM1_2.1 (+)	
qTL.3E.H6.1 (+)	qTL_3.1 (+)		
qTL4.H6.1 (+)			qFL4A.H6.1 (-)
qTL4.H6.2 (+)			qFL4D.H6.1 (+)
qTL.6A.H6.1 (+)	qTL_6.1 (+)	qM1_6.1 (+)	
qTL.6B.H6.1 (-)	qTL_6.1 (+)	qM1_6.1 (+)	qFL6B.H6.2 (+)
qTL.9B.H6.1 (+)			
qTL.10C.H6.1 (-)			qFL10A.H6.1 (-)
qTL10.H6.2 (+)			qFL10.H6.1 (+)

Similarly, a total of two and five QTLs for **BRCH** show possible correspondence to **FL** in the H4 and H6-derived BC_1_F_2_ populations, respectively (Table 4), with four pairs of overlapping QTLs showing opposite allele effect from *S*. *halepense*. Additional QTLs that overlap but are not limited to the same population are qBRCH.4C.H4.1 and qFL4A H6.1, peaking at 4.8 and 4.3 Mb respectively; and qBRCH4D.H4.1 and qFL4D.H6.1, peaking at 61.2 and 62.8 Mb, respectively. QTLs qBRCH10C.H6.1/2 and qFL10A.H6.1 might be loosely associated, since they both cover a large genomic region.

**Table 4:** Comparisons of BRCH QTL in SBSH-BC_1_F_2_, IS-RIL and PQ-RIL populations.

QTL Name	ISRIL	PQRIL	FLQTL
qBRCH1.H4.2 (+)		qAX1.1 (+)	
qBRCH1.H4.1 (+)		qAX1.1 (+)	qFL.1A.H4.1 (-)
qBRCH.1F.H4.1 (-)			
qBRCH.2D.H4.1 (-)			
qBRCH3.H4.1 (+)		qAX3.1, qIM3.1 qVG3.1, qSR3.1, qTR3.1 (+)	
qBRCH.4C.H4.1 (-)			
qBRCH4.H4.1 (+)			
qBRCH.4D.H4.1 (-)	qBRCH4.1 (-)	qBRCH4.1 (-)	
qBRCH.5C.H4.1 (-)	qBRCH5.1 (+)	qTR5.1 (-)	
qBRCH.6B.H4.1 (-)			qFL.6B.H4.1 (+)
qBRCH6.H4.2 (+)			
qBRCH.7C.H4.1 (+)			
qBRCH9.H4.1 (+)			
qBRCH10.H4.1 (+)			
qBRCH.1C.H6.1 (+)		qIM2_1.1 (+)	
qBRCH.3E.H6.1 (+)		qAX3.1, qIM3.1, qVG3.1, qSR3.1, qTR3.1 (+)	
qBRCH3.H6.2 (+)			
qBRCH4.H6.1 (+)	qBRCH4.2 (+)		qFL.4D.H6.1 (+)
qBRCH5.H6.1 (+)			
qBRCH.5C.H6.1 (+)			
qBRCH.6B.H6.1 (-)			qFL.6B.H6.1 (+)
qBRCH.6B.H6.2 (-)			qFL.6B.H6.2 (+)
qBRCH6.H6.1 (+)			qFL.6B.H6.2 (+)
qBRCH6.H6.3 (+)			qFL.6E.H6.1 (+)
qBRCH7.H6.1 (+)		qVG7.1 (+), qIM2_7.1 (+), qSR_7.1 (-)	
qBRCH9.H6.1 (+)		qTR9.1 (+)	
qBRCH.10C.H6.1 (+)	qBRCH10.1 (-)		
qBRCH.10C.H6.2 (-)	qBRCH10.1 (-)		

### Comparison to QTLs found in IS-RIL and PQ-RIL

We found a total of five **TL** QTLs in the SH-BC_1_F_2_, qTL.1D.H6.1, qTL.3E.H6.1, qTL.6A.H6.1, qTL.6B.H6.1, and qTL7.H4.1, corresponding in physical position on the sorghum genome sequence to four IS-RIL **TL** QTLs, qTL_1.1, qTL_3.1, qTL_6.1 and qTL7.1; and a total of seven SBSH-BC_1_F_2_ **TL** QTLs corresponding to five PQ-RIL QTLs (Table 3). Curiously, qTL.6A.H6.1 and qTL.6B.H6.1, not overlapping with each other but both overlapping with QTLs found in the IS-RIL and PQ-RIL populations, display opposite allele effects. qTL.6B.H6.1 from the SH-BC_1_F_2_ population shows a negative effect, indicating that fewer tillers may be associated with late flowering, which might be associated with the *Ma1* [18] gene on chromosome 6. However, there appears to be another QTL region on chromosome 6 significant in all three populations, roughly spanning 50–60 Mb, and suggesting that *S*. *bicolor* alleles reduce the number of tillers.

Despite that **BRCH** is a plastic trait with low heritability, we still found two **BRCH** QTLs in the H4-derived SBSH-BC_1_F_2_, qBRCH4D.H4.1 and qBRCH5C.H4.1 overlapping with two IS-RIL QTLs, qRBCH4.1 and qBRCH5.1; and three H6-derived SBSH-BC_1_F_2_ QTLs overlapping with two IS-RIL QTLs, qBRCH4.2 and qBRCH10.1 (Table 4). Two pairs of QTLs, qBRCH.5C.H4.1 and qBRCH5.1 from IS-RIL and qBRCH.10C.H6.1 and qBRCH10.1 from IS-RIL show opposite allele effects from *S*. *halepense*, suggesting that *S*. *bicolor* alleles increase BRCH in the SBSH-BC_1_F_2_ population but decrease it in the ISRIL. This implies that IS3620c has an allele conferring abundant branching, with the BTx623 allele conferring less branching than IS3620c but more than *S*. *halepense*. In addition, a total of five and four H4 and H6–derived SBSH-BC_1_F_2_ QTLs for **BRCH** overlap with QTLs for various degrees (primary, secondary or tertiary) of vegetative branching in PQRIL described in Kong, Guo [6]. Most overlapping pairs of QTLs of SBSH-BC_1_F_2_ and the PQRIL show the same direction of effect, from *S*. *halepense* and *S*. *propinquum*, respectively, except one case on chromosome 7 where QTLs within PQRIL shows different directions of effects (Table 4; within the PQRIL population, qSR_7.1 showed negative effect while qVG7.1 and qIM2_7.1 showed positive effect).

### A regression model to predict biomass

We performed a regression analysis to predict biomass weight (**Biomass**, using natural log transformation) with respect to traits related to plant architecture while controlling for population structure and environmental factors. Our final model consists of a total of seven variables, with plant height (**PH**), mid-stalk diameter (**MD**), number of mature tillers (**TL**), number of secondary branches (**BRCH**), flowering time (**FL**), and population (**H4** or **H6**) as fixed effects and environmental factors as a random effect (Table 5). Fixed effects (the environmental factor) in this model collectively explain about 71.76% of the total variance using a modified method for estimating R-squared in mixed models [36]. The typical log error in this model is about 0.3148, and can be decomposed into environmental error that is estimated to be normally distributed with a mean of zero and standard deviation of 0.1260; and the inherent residual error that is estimated to be normally distributed with a mean of zero and standard deviation of 0.2885 (Table 5A). The model suggests that **PH**, **TL** and **MD** are the three most important variables in predicting **Biomass**, followed by **FL** and **BRCH** (Table 5B). For example, a 10 cm increase in plant height leads to 6.4% increase in **Biomass** weight, keeping other variables constant, while an increase of one TL leads to a 15.1% increase in Biomass weight, keeping other variables constant.

**Table 5:** A mixed-effect model and parameter estimations for predicting Biomass (natural log transformation) in the SBSH-BC_1_F_2_ population.

(a)Variance components					
Groups		Variance		Std. dev.	
Env		0.01589		0.1260	
Residual		0.08324		0.2885	
(b)Modeling					
	Sum Sq	Mean Sq	DF	F-stat	P value
**PH**	32.561	32.561	1	391.19	< 2.2e-16 ***
**MD**	10.609	10.609	1	127.46	< 2.2e-16 ***
**TL**	25.421	25.421	1	305.42	< 2.2e-16 ***
**BRCH**	2.303	2.303	1	27.67	1.901e-07 ***
**FL**	4.892	4.892	1	58.77	4.396e-14 ***
Population	1.944	1.944	1	23.36	1.564e-06 ***
(c)Parameter estimation					
	Estimate	Std. Error	df	t-stat	P-value
(Intercept)	2.6460	0.1066	19.8	24.815	2.22E-16***
**PH**	0.006197	0.000313	936.3	19.779	< 2e-16***
**MD**	0.02962	0.002624	953.4	11.290	< 2e-16***
**TL**	0.1409	0.008061	675.1	17.476	< 2e-16***
**BRCH**	0.06978	0.01327	720.6	5.260	1.9E-07***
**FL**	0.007882	0.001028	942.7	7.666	4.4E-14***
Population H6	-0.09472	0.01960	954.8	-4.833	1.56E-06***

PH: Plant height.

MD: Mid-stalk diameter.

TL: Number of mature tillers.

BRCH: Number of secondary branches per tiller.

Env: Environmental effects.

## Discussion

The present study offers several new insights into the genetic control of tillering and vegetative branching. First, it adds more information to current knowledge of vegetative branching in sorghum, an under-studied trait, especially providing early insights into QTL polymorphism in *S*. *halepense*. Correspondence of QTL regions between three populations sharing *S*. *bicolor* BTx623 as a common parent, with the other parents being morphologically and genetically distinct genotypes that represent cultivated (IS3620C), wild diploid (*S*. *propinquum*) and wild polyploid (*S halepense*) sorghums, provides information about common QTLs shared between or among populations and taxon-specific QTLs that contribute to divergence. Finally, constructing a mixed model to predict dry biomass with respect to various traits associated with plant architecture and the environmental factors provides a framework to prioritize each trait in selection for biomass, as well as quantifying environmental influences.

### QTL mapping

QTL mapping results for two relatively plastic traits, **TL** and **BRCH**, suggest high genotype by environment interactions and population differences. We only found three and one QTLs significant in multiple environments for **TL** and **BRCH** with interval mapping, respectively, with 6 and 13 significant in only single environments. Overlapping SNP sets from single marker analysis are much lower for these traits than for highly heritable traits such as plant height and flowering time (S1 and S2 Figs).

QTL results are very different in the two populations derived from two different sibling *S*. *halepense* x *S*. *bicolor* F_1_ plants, possibly due to *Ma* and *Dw* genes on chromosome 6. We detected fewer **TL** QTLs in the H4 than the H6-derived population, as was also true of **FL** and **PH** QTLs [30]. The number of **BRCH** QTLs for the two populations does not follow this pattern, but most H4-derived QTLs had negative effects in interval mapping, indicating the *S*. *halepense* allele reduced **BRCH**. Unexpected cases in which *S*. *halepense* alleles reduce **TL** are associated with **FL** QTLs, qTL4.H6.1 and qFL4A.H6.1, qTL.6B.H6.1 and qFL6B.H6.2; and *S*. *halepense* alleles that reduce **BRCH** associated with **FL** QTLs, qBRCH1.H4.1 and qFL.1A.H4.1, qBRCH.6B.H4.1, and qFL.6B.H4.1, qBRCH.6B.H6.1 and qFL.6B.H6.1, and qBRCH.6B.H6.2 and qFL.6B.H6.2 (Tables 3 and 4). This finding suggests that delaying flowering might reduce tillers and branching, perhaps due to late release of apical dominance.

### QTL correspondence

Two **TL** QTLs and one **BRCH** QTL overlapped in all three populations (this study, ISRIL and PQRIL) with the same direction of allele effect (Tables 3 and 4), suggesting a parsimonious hypothesis that *S*. *halepense*, *S*. *propinquum* and *S*. *bicolor* IS3620C share an ancestral allele, while a different recently-derived allele has been selected in the elite cultivar *S*. *bicolor* BTx623. Cases in which overlapping QTLs have different directions of allele effect are more complex, possibly suggesting more than two alleles, or perhaps representing spurious correspondence due to relatively large QTL intervals.

The *S*. *halepense* data continue to support the hypothesis that **TL** and **BRCH** are developmentally related [6]—six QTL pairs (qTL2.H4.1 and qBRCH.2D.H4.1, qTL.4D.H4.1 and qBRCH.4D.H4.1,qTL.3E.H6.1 and qBRCH.3E.H6.1, qTL.6B.H6.1 and qRBCH.6B.H6.2, qTL6A.H6.1 and qBRCH6.H6.3, qTL.10C.H6.1 and qBRCH.10C.H6.2) overlapped, perhaps harboring genes influencing axillary meristem development at early stages.

**Fig 5:**
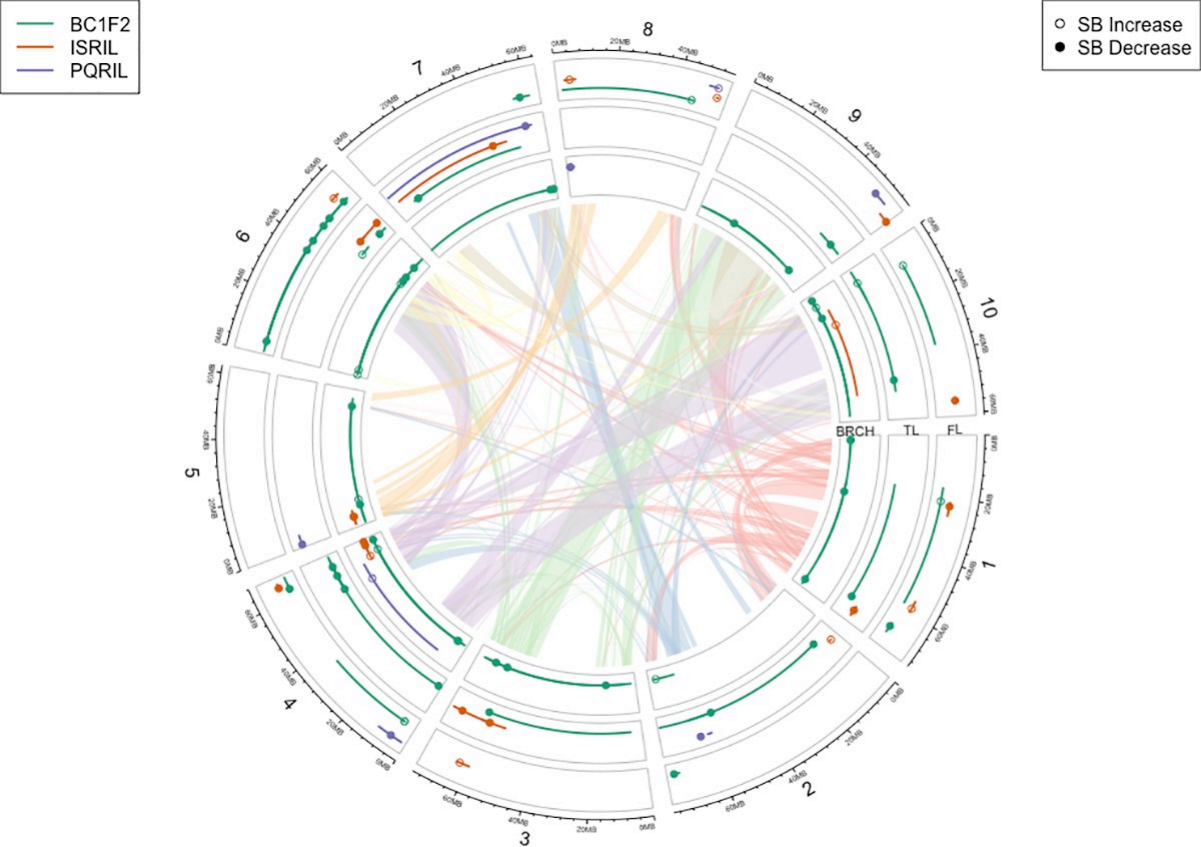
QTL correspondence for flowering time (FL), tillering (TL), and secondary branching (BRCH) in SBSH-BC_1_F_2_, ISRIL and PQRIL populations in physical distance. Links are the duplication events in sorghum [42].

A surprising number of genomic regions were significant for **FL** and **TL** or **FL** and **BRCH**, perhaps suggesting pleiotropic relationships (Tables 3 and 4, Fig 5). For example, genes regulating flowering such as MADS box proteins also influence determinacy of other meristems [39]. Further, the flowering locus T (*FT*) gene that regulates flowering time in many species, has recently been found to trigger storage organ formation through direct interaction with the TCP factors [13]. We found a total of six genomic regions harboring QTLs responsible for both **FL** and **TL**, and four regions for both **FL** and **BRCH** in their respective populations. Previous study [21,27,40] has suggested that regions on chromosome 6 that harbor *Ma1* also contain QTLs for tiller number. One explanation might be that *Ma1*, which appears to be a homolog of the Arabidopsis *Ft* and Rice *Hd3a* genes [18], influences organ formation. The *Ma1* associated region in this study affected both **TL** and **BRCH**, while another QTL region at ~47.2Mb on chromosome 6 affecting all three traits, **FL**, **TL** and **BRCH**. This QTL (~47.2Mb) might be related to the Sb06g019010 or (Sobic.006G107400.1 in *Sorghum bicolor* v3.11) gene encoding the ‘number of apical meristem’ (NAM) protein [6,41].

### Regression model for predicting biomass

Plant architecture related traits can predict **Biomass** with relatively high accuracy. A mixed model for predicting dry biomass weight (**Biomass**) retained a total of five traits, plant height (**PH**), mid-stalk diameter (**MD**), mature tillers (**TL**), number of secondary branches (**BRCH**), and flowering time (**FL**) as significant predictors of dry biomass. The fixed effects explains 71.76% of the total variance, and a log error of 0.3148. Application of this model might be a cost-efficient method for predicting **Biomass** for future experiments, quantifying the contribution of individual traits to **Biomass** and providing guidance for improving genotypes aimed at biomass production.

## Supporting information

S1 Fig Venn diagram of the number of SNP markers for tillering (TL) significant at a p value<10^−3^ in different environments for pooled SBSH BC_1_F_2_ populations. (DOCX)

S2 Fig Venn diagram of the number of SNP markers for secondary branches per tiller (BRCH) significant at a pvalue<10^−3^ in different environments for SBSH BC_1_F_2_ pooled populations. (DOCX)

S1 Table Summary statistics for number of mature tillers (TL) and number of secondary branches (BRCH) in the SBSH-BC_1_F_2_ [*S*. *halepense* derived (*S*. *bicolor* BTx623× *S*. *halepense* G9E) backcross] population and parents. (DOCX)

S2 Table Summary statistics for the number of mature tillers (TL) and number of secondary branches (BRCH) in the PQ-RIL [propinquum derived (*S*. *bicolor* BTx623× *S*. *propinquum*) recombinant inbred line] population and parents. (DOCX)

S3 Table Summary statistics for number of mature tillers (TL) and number of secondary branches (BRCH) in the IS-RIL [IS3620C derived (*S*. *bicolor* BTx623× *S*. *bicolor* IS3620C) recombinant inbred line] population and parents. (DOCX)

S4 Table Parameters of tillering (TL) QTLs from single marker analysis of the H4 and H6 SBSH-BC_1_F_2_ populations. (DOCX)

S5 Table Parameters of number of secondary branches per tiller (BRCH) QTLs from single marker analysis of the H4 and H6 SBSH-BC_1_F_2_ populations. (DOCX)

S6 Table Parameters of tillering and vegetative branching related QTLs from interval mapping of the IS-RIL population. (DOCX)

S7 Table Parameters of plant architecture related QTLs from interval mapping of the PQ-RIL population. (DOCX)

S1 File Phenotype of the H4 population. (XLSX)

S2 File Phenotype of the H6 population. (XLSX)
