# Supplementary material for: Comparative evolution of vegetative branching in sorghum
Source: PLoS One. 2021 Aug 13;16(8):e0255922. doi: 10.1371/journal.pone.0255922 (PMC8362987; doi:10.1371/journal.pone.0255922)

S1 Fig. Venn diagram of the number of SNP markers for tillering (TL) significant at a P-value<10^-3^ in different environments for pooled SBSH BC_1_F_2_ populations


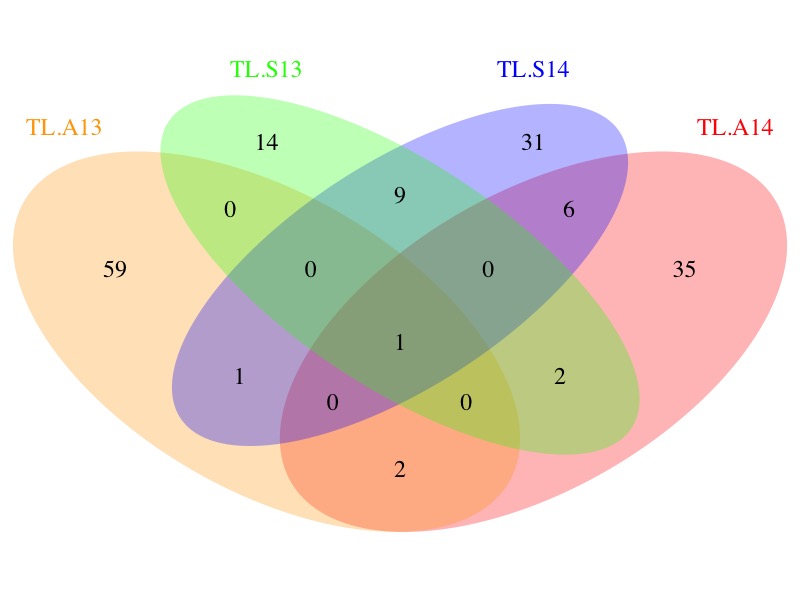

Supplement: S1 Fig — (DOCX) [file pone.0255922.s001.docx]
