# Supplementary material for: Comparative evolution of vegetative branching in sorghum
Source: PLoS One. 2021 Aug 13;16(8):e0255922. doi: 10.1371/journal.pone.0255922 (PMC8362987; doi:10.1371/journal.pone.0255922)

S2 Fig. Venn diagram of the number of SNP markers for secondary branches per tiller (BRCH) significant at a P-value<10^-3^ in different environments for SBSH BC_1_F_2_ pooled populations


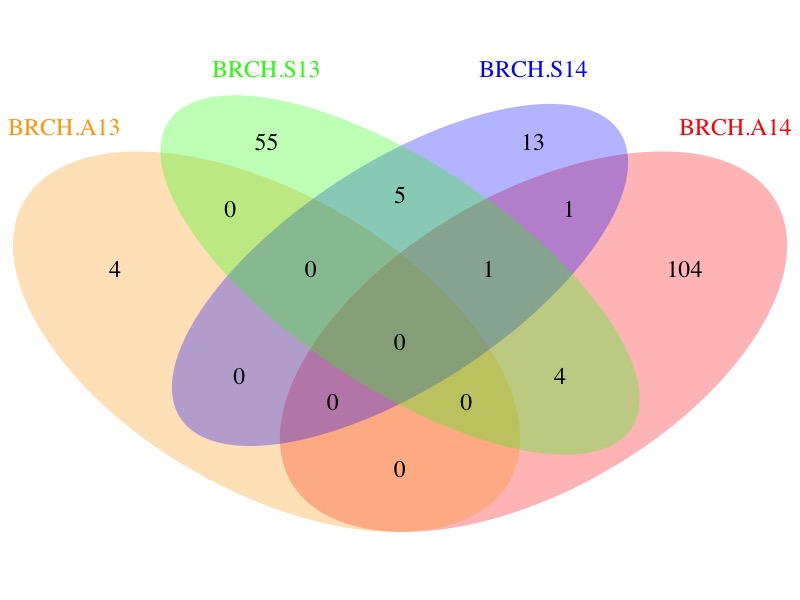

Supplement: S2 Fig — (DOCX) [file pone.0255922.s002.docx]
