# Supplementary material for: Comparative evolution of vegetative branching in sorghum
Source: PLoS One. 2021 Aug 13;16(8):e0255922. doi: 10.1371/journal.pone.0255922 (PMC8362987; doi:10.1371/journal.pone.0255922)
Supplement: S1 Table — (DOCX) [file pone.0255922.s003.docx]

Table S1 Summary statistics for number of mature tillers (TL) and number of secondary branches (BRCH) in the SBSH-BC_1_F_2_ [*S. halepense* derived (*S. bicolor* BTx623× *S. halepense* G9E) backcross] population and parents.

|  |  |  | SH-BC1F2 | | | | | | | BTx623 (2x) | | | BTx623 (4x) | | |  |
| --- | --- | --- | --- | --- | --- | --- | --- | --- | --- | --- | --- | --- | --- | --- | --- | --- |
| Trait | Location | Year | Population | N | Mean | Median | SD | Min | Max | N | Mean | SD | N | Mean | SD | Heritability (%) |
| TL | Athens | 2013 | Pooled | 246 | 2.43 | 2.17 | 0.9303 | 0.83 | 5.33 | 12 | 1.3 | 0.49 | 18 | 1.9 | 1.39 | 30.46 |
| TL | Athens | 2013 | H4 | 141 | 2.29 | 2.17 | 0.8410 | 0.83 | 5.33 |  |  |  |  |  |  |  |
| TL | Athens | 2013 | H6 | 105 | 2.61 | 2.50 | 1.0140 | 1.17 | 5.17 |  |  |  |  |  |  |  |
| TL | Athens | 2014 | Pooled | 236 | 4.66 | 4.67 | 1.2782 | 2.00 | 8.75 | 8 | 2.0 | 0.87 | 12 | 3.7 | 1.03 |  |
| TL | Athens | 2014 | H4 | 134 | 4.63 | 4.67 | 1.1948 | 2.00 | 8.67 |  |  |  |  |  |  |  |
| TL | Athens | 2014 | H6 | 102 | 4.71 | 4.58 | 1.3849 | 2.17 | 8.75 |  |  |  |  |  |  |  |
| TL | Salina | 2013 | Pooled | 246 | 3.92 | 3.83 | 1.0259 | 1.67 | 7.50 | 12 | 1.3 | 0.45 | 12 | 2.2 | 1.19 |  |
| TL | Salina | 2013 | H4 | 141 | 3.83 | 3.67 | 1.0840 | 2.00 | 7.50 |  |  |  |  |  |  |  |
| TL | Salina | 2013 | H6 | 105 | 4.04 | 4.00 | 0.9346 | 1.67 | 6.67 |  |  |  |  |  |  |  |
| TL | Salina | 2014 | Pooled | 236 | 6.09 | 5.75 | 1.4246 | 3.50 | 12.00 | 12 | 3.3 | 1.48 | 12 | 5.0 | 1.08 |  |
| TL | Salina | 2014 | H4 | 134 | 5.96 | 5.67 | 1.3826 | 4.00 | 12.00 |  |  |  |  |  |  |  |
| TL | Salina | 2014 | H6 | 102 | 6.26 | 6.00 | 1.4672 | 3.50 | 11.00 |  |  |  |  |  |  |  |
| BRCH | Athens | 2013 | Pooled | 246 | 2.79 | 2.71 | 0.7409 | 1.25 | 5.42 | 12 | 1.6 | 1.03 | 18 | 3.7 | 1.15 | 10.38 |
| BRCH | Athens | 2013 | H4 | 141 | 2.74 | 2.67 | 0.6851 | 1.25 | 4.70 |  |  |  |  |  |  |  |
| BRCH | Athens | 2013 | H6 | 105 | 2.87 | 2.75 | 0.8072 | 1.25 | 5.42 |  |  |  |  |  |  |  |
| BRCH | Athens | 2014 | Pooled | 236 | 2.34 | 2.33 | 0.5415 | 0.78 | 3.83 | 12 | 2.4 | 0.79 | 12 | 1.1 | 0.83 |  |
| BRCH | Athens | 2014 | H4 | 134 | 2.27 | 2.25 | 0.5517 | 0.78 | 3.67 |  |  |  |  |  |  |  |
| BRCH | Athens | 2014 | H6 | 102 | 2.42 | 2.42 | 0.5177 | 1.33 | 3.83 |  |  |  |  |  |  |  |
| BRCH | Salina | 2013 | Pooled | 246 | 1.57 | 1.50 | 0.8560 | 0.00 | 4.67 | 12 | 0.1 | 0.29 | 12 | 0.1 | 0.29 |  |
| BRCH | Salina | 2013 | H4 | 141 | 1.31 | 1.17 | 0.8050 | 0.00 | 4.67 |  |  |  |  |  |  |  |
| BRCH | Salina | 2013 | H6 | 105 | 1.91 | 1.83 | 0.8036 | 0.50 | 4.00 |  |  |  |  |  |  |  |
| BRCH | Salina | 2014 | Pooled | 236 | 0.97 | 0.83 | 0.7857 | 0.00 | 3.50 | 12 | 0.3 | 1.11 | 12 | 0.0 | 0.00 |  |
| BRCH | Salina | 2014 | H4 | 134 | 0.71 | 0.63 | 0.5858 | 0.00 | 2.67 |  |  |  |  |  |  |  |
| BRCH | Salina | 2014 | H6 | 102 | 1.32 | 1.17 | 0.8800 | 0.00 | 3.50 |  |  |  |  |  |  |  |
