# Supplementary material for: Comparative evolution of vegetative branching in sorghum
Source: PLoS One. 2021 Aug 13;16(8):e0255922. doi: 10.1371/journal.pone.0255922 (PMC8362987; doi:10.1371/journal.pone.0255922)
Supplement: S2 Table — (DOCX) [file pone.0255922.s004.docx]

Table S2 Summary statistics for the number of mature tillers (**TL**) and number of secondary branches (**BRCH**) in the PQ-RIL [propinquum derived (*S. bicolor* BTx623× *S. propinquum*) recombinant inbred line] population and parents.

|  |  | PQ-RIL | |  |  |  |  | BTx623 | |  |  |
| --- | --- | --- | --- | --- | --- | --- | --- | --- | --- | --- | --- |
| Trait | Year | N | Mean | Median | SD | Min | Max | N | Mean | SD | Heritability (%) |
| TL | 2009 | 155 | 3.11 | 3.00 | 1.8495 | 1.0 | 11.0 | 10 | 1.20 | 0.4216 | 35.17 |
| TL | 2010 | 132 | 4.79 | 4.50 | 2.8673 | 1.0 | 14.5 | 14 | 1.21 | 0.4472 |  |
| TL | 2011 | 141 | 4.08 | 3.50 | 2.1624 | 1.0 | 13.5 | 20 | 1.65 | 0.8127 |  |
| BRCH | 2009 | 155 | 4.34 | 3.67 | 2.6632 | 0.2 | 16.0 | 10 | 3.25 | 1.0341 | 10.38 |
| BRCH | 2010 | 132 | 4.70 | 4.00 | 2.5093 | 0.0 | 15.6 | 14 | 3.47 | 1.2175 |  |
| BRCH | 2011 | 141 | 3.60 | 3.43 | 1.7645 | 0.0 | 10.7 | 20 | 1.83 | 1.4754 |  |
