# Supplementary material for: Comparative evolution of vegetative branching in sorghum
Source: PLoS One. 2021 Aug 13;16(8):e0255922. doi: 10.1371/journal.pone.0255922 (PMC8362987; doi:10.1371/journal.pone.0255922)
Supplement: S3 Table — (DOCX) [file pone.0255922.s005.docx]

Table S3 Summary statistics for number of mature tillers (**TL**) and number of secondary branches (**BRCH**) in the IS-RIL [IS3620C derived (*S. bicolor* BTx623× *S. bicolor* IS3620C) recombinant inbred line] population and parents.

|  | IS-RIL | | | | | | | BTx623 | | | IS3620C | | |  |
| --- | --- | --- | --- | --- | --- | --- | --- | --- | --- | --- | --- | --- | --- | --- |
| Trait | Year | N | Mean | Median | SD | Min | Max | N | Mean | SD | N | Mean | SD | Heritability (%) |
| TL | 2011 | 388 | 2.29 | 2.00 | 1.2250 | 1.00 | 10.00 | 16 | 1.56 | 0.8638 | 13 | 5.23 | 1.9277 | 36.09 |
| TL | 2012 | 384 | 2.58 | 2.50 | 1.2970 | 1.00 | 10.00 | 12 | 1.25 | 0.4330 | 16 | 4.00 | 1.7678 |  |
| BRCH | 2011 | 388 | 2.63 | 2.50 | 1.7218 | 0.00 | 10.50 | 16 | 3.31 | 1.7399 | 13 | 5.03 | 3.2963 | 40.92 |
| BRCH | 2012 | 383 | 2.90 | 2.80 | 1.5127 | 0.00 | 13.50 | 12 | 4.63 | 1.9378 | 16 | 2.89 | 0.8393 |  |
