# Supplementary material for: Comparative evolution of vegetative branching in sorghum
Source: PLoS One. 2021 Aug 13;16(8):e0255922. doi: 10.1371/journal.pone.0255922 (PMC8362987; doi:10.1371/journal.pone.0255922)
Supplement: S4 Table — (DOCX) [file pone.0255922.s006.docx]

Table S4 Parameters of tillering (**TL**) QTLs from single marker analysis of the H4 and H6 SBSH-BC_1_F_2_ populations

| QTL | Peak SNP | Chr | Pos | P vlaue | Effect | Left ^1^ | Right^1^ |
| --- | --- | --- | --- | --- | --- | --- | --- |
| qTL2.H4.1 | S2_56654701 | 2 | 56.7 | 3.16E-06 | 1.44 | S2_56654701 | S2_77026507 |
| qTL4.H4.1 | S4_53529686 | 4 | 53.5 | 0.000103577 | 0.95 | S4_52973418 | S4_61151050 |
| qTL4.H4.2 | S4_57873900 | 4 | 57.9 | 6.20E-05 | 1.24 | S4_57041432 | S4_62529365 |
| qTL7.H4.1 | S7_9441761 | 7 | 9.4 | 0.000282094 | 1.09 | S7_7725211 | S7_53456010 |
| qTL1.H6.1 | S1_62664534 | 1 | 62.7 | 3.78E-05 | 2.10 | S1_5081943 | S1_70788904 |
| qTL1.H6.2 | S1_67465078 | 1 | 67.5 | 5.19E-05 | 1.18 | S1_1265914 | S1_69679431 |
| qTL2.H6.1 | S2_7106903 | 2 | 7.1 | 2.61E-05 | 0.90 | S2_6849865 | S2_69967980 |
| qTL3.H6.1 | S3_15352565 | 3 | 15.4 | 0.000180592 | 1.07 | S3_6850696 | S3_66467840 |
| qTL3.H6.2 | S3_57375930 | 3 | 57.4 | 3.12E-05 | 1.30 | S3_6871352 | S3_72811253 |
| qTL4.H6.1 | S4_1267007 | 4 | 1.3 | 0.000120412 | 1.58 | S4_1051006 | S4_67106558 |
| qTL4.H6.2 | S4_59173211 | 4 | 59.2 | 4.34E-06 | 1.53 | S4_55435164 | S4_62067548 |
| qTL6.H6.1 | S6_50834320 | 6 | 50.8 | 0.000142023 | 1.91 | S6_45819310 | S6_61351403 |
| qTL6.H6.2 | S6_50892527 | 6 | 50.9 | 8.30E-07 | -1.06 | S6_941772 | S6_51751708 |
| qTL6.H6.3 | S6_61374763 | 6 | 61.4 | 7.32E-05 | 0.93 | S6_56308299 | S6_61939440 |
| qTL9.H6.1 | S9_53370872 | 9 | 53.4 | 1.71E-05 | 0.99 | S9_8217569 | S9_58139966 |
| qTL9.H6.2 | S9_56158950 | 9 | 56.2 | 0.000119584 | 1.52 | S9_51954880 | S9_57982011 |
| qTL10.H6.1 | S10_6764501 | 10 | 6.8 | 0.000158086 | -1.26 | S10_551695 | S10_47850983 |
| qTL10.H6.2 | S10_45853249 | 10 | 45.9 | 8.56E-05 | 1.48 | S10_5531584 | S10_50040659 |

^1^ Markers with smallest/largest (left/right) physical distances within in an interval
