# Supplementary material for: Comparative evolution of vegetative branching in sorghum
Source: PLoS One. 2021 Aug 13;16(8):e0255922. doi: 10.1371/journal.pone.0255922 (PMC8362987; doi:10.1371/journal.pone.0255922)
Supplement: S5 Table — (DOCX) [file pone.0255922.s007.docx]

Table S5 Parameters of number of secondary branches per tiller (**BRCH**) QTLs from single marker analysis of the H4 and H6 SBSH-BC_1_F_2_ populations

| QTL | | Peak SNP | Chr | Pos | P vlaue | Effect | Left | Right |
| --- | --- | --- | --- | --- | --- | --- | --- | --- |
| qBRCH1.H4.1 | S1_2170283 | | 1 | 2.2 | 1.96E-05 | 0.44 | S1_738323 | S1_70832264 |
| qBRCH1.H4.2 | S1_25951730 | | 1 | 26.0 | 8.89E-06 | 0.83 | S1_21843308 | S1_58839105 |
| qBRCH3.H4.1 | S3_13576771 | | 3 | 13.6 | 4.72E-06 | 0.55 | S3_2231352 | S3_69864145 |
| qBRCH4.H4.1 | S4_5197267 | | 4 | 5.2 | 4.09E-05 | 0.66 | S4_2665334 | S4_65162214 |
| qBRCH4.H4.2 | S4_68004088 | | 4 | 68.0 | 8.23E-06 | -0.58 | S4_4826988 | S4_68004088 |
| qBRCH5.H4.1 | S5_11626618 | | 5 | 11.6 | 9.53E-08 | -0.55 | S5_2287684 | S5_56190491 |
| qBRCH6.H4.1 | S6_941772 | | 6 | 0.9 | 4.45E-05 | -0.59 | S6_941772 | S6_58837002 |
| qBRCH6.H4.2 | S6_50891801 | | 6 | 50.9 | 6.19E-05 | 0.48 | S6_48190030 | S6_59129413 |
| qBRCH7.H4.1 | S7_61629178 | | 7 | 61.6 | 0.000126756 | 0.39 | S7_887957 | S7_62553133 |
| qBRCH9.H4.1 | S9_50415229 | | 9 | 50.4 | 0.00011769 | 0.62 | S9_4506578 | S9_50415229 |
| qBRCH10.H4.1 | S10_11562619 | | 10 | 11.6 | 4.03E-05 | 0.71 | S10_2754555 | S10_53317886 |
|  |  | |  |  |  |  |  |  |
| qBRCH1.H6.1 | S1_70199577 | | 1 | 70.2 | 1.72E-05 | 0.44 | S1_69679431 | S1_72932488 |
| qBRCH3.H6.1 | S3_61786995 | | 3 | 61.8 | 4.93E-05 | 1.10 | S3_4015390 | S3_71069563 |
| qBRCH3.H6.2 | S3_65327695 | | 3 | 65.3 | 3.28E-05 | 0.55 | S3_61695348 | S3_70980529 |
| qBRCH4.H6.1 | S4_66003764 | | 4 | 66.0 | 0.000129728 | 1.42 | S4_1267007 | S4_66003764 |
| qBRCH5.H6.1 | S5_9191183 | | 5 | 9.2 | 1.82E-05 | 1.25 | S5_791861 | S5_54509359 |
| qBRCH6.H6.1 | S6_48740921 | | 6 | 48.7 | 2.01E-05 | 1.40 | S6_19018634 | S6_58002893 |
| qBRCH6.H6.2 | S6_49144952 | | 6 | 49.1 | 0.000135526 | -0.67 | S6_3185305 | S6_51252650 |
| qBRCH6.H6.2 | S6_56567550 | | 6 | 56.6 | 0.00059907 | 0.76 | S6_56567550 | S6_61939440 |
| qBRCH7.H6.1 | S7_63121928 | | 7 | 63.1 | 4.14E-05 | 1.08 | S7_42048 | S7_63121928 |
| qBRCH9.H6.1 | S9_17340892 | | 9 | 17.3 | 4.44E-05 | 0.99 | S9_825289 | S9_39917345 |
| qBRCH10.H6.1 | S10_15907044 | | 10 | 15.9 | 5.11E-06 | 1.24 | S10_2406347 | S10_57704601 |
