# Supplementary material for: Comparative evolution of vegetative branching in sorghum
Source: PLoS One. 2021 Aug 13;16(8):e0255922. doi: 10.1371/journal.pone.0255922 (PMC8362987; doi:10.1371/journal.pone.0255922)
Supplement: S6 Table — (DOCX) [file pone.0255922.s008.docx]

Table S6 Parameters of tillering and vegetative branching related QTLs from interval mapping of the IS-RIL population

| QTL | Year | Peak (cM) | Peak (Mb) | LOD | % Var | Additive Effect | Left Flanking SNP Positions^1^ | Right Flanking SNP Positions |
| --- | --- | --- | --- | --- | --- | --- | --- | --- |
| qTL_1.1 | 2012 | 171.0 | 69.2 | 1.9 | 2.96 | 0.0695 | 67.7 | 71.3 |
| qTL_2.1 | 2011 | 6.0 | 3.2 | 5.1 | 5.98 | -0.0946 | 3.1 | 3.8 |
| qTL_3.1 | 2011 | 98.0 | 58.1 | 3.8 | 3.65 | 0.0758 | 52.0 | 60.9 |
| qTL_3.2 | 2012 | 143.0 | 69.2 | 2.7 | 3.40 | 0.0787 | 58.1 | 72.7 |
| qTL_6.1 | 2012 | 55.0 | 50.6 | 3.2 | 2.47 | 0.0638 | 49.6 | 53.0 |
| qTL_6.1 | 2011 | 102.0 | 59.8 | 3.5 | 2.16 | 0.0593 | 49.6 | 60.9 |
| qTL_7.1 | 2012 | 27.0 | 43.8 | 3.5 | 3.55 | 0.0764 | 3.1 | 49.0 |
|  |  |  |  |  |  |  |  |  |
| qBRCH4.1 | 2012 | 121.6 | 60.0 | 3.0 | 4.04 | -0.1049 | 58.9 | 65.8 |
| qBRCH4.2 | 2012 | 153.6 | 65.2 | 1.1 | 2.77 | 0.0916 | 63.1 | 65.8 |
| qBRCH4.2 | 2011 | 162.0 | 66.9 | 3.5 | 3.53 | 0.1203 | 65.8 | 67.6 |
| qBRCH5.1 | 2011 | 30.6 | 4.8 | 4.6 | 4.70 | 0.1350 | 1.5 | 7.4 |
| qBRCH5.1 | 2012 | 30.0 | 4.8 | 4.3 | 5.37 | 0.1241 | 1.5 | 6.1 |
| qBRCH10.1 | 2012 | 57.8 | 17.0 | 3.3 | 3.71 | -0.0982 | 9.8 | 49.4 |

^1^ SNP positions correspond to 1-lod QTL interval
