# Supplementary material for: Comparative evolution of vegetative branching in sorghum
Source: PLoS One. 2021 Aug 13;16(8):e0255922. doi: 10.1371/journal.pone.0255922 (PMC8362987; doi:10.1371/journal.pone.0255922)
Supplement: S7 Table — (DOCX) [file pone.0255922.s009.docx]

Table S7 Parameters of plant architecture related QTLs from interval mapping of the PQ-RIL population

| QTL | Env | Peak (cM) | Peak (Mb) | LOD | % Var | Additive Effect | Left Flanking Marker (Mb) | Right Flanking Marker (Mb) |
| --- | --- | --- | --- | --- | --- | --- | --- | --- |
| qTL_2.1 | 2010 | 55.9 | 63.2 | 3.9 | 12.66 | 1.30 | 59.1 | 60.5 |
| qTL_5.1 | 2011 | 6.0 | 0.2-1.9 | 2.7 | 5.77 | 0.70 | 0.2 | 4.5 |
| qTL_7.1 | 2009 | 40.0 | 57.3 | 2.5 | 7.12 | 0.14 | 0.9 | 59.4 |
|  |  |  |  |  |  |  |  |  |
| qBRCH4.1 | 2011 | 48.0 | 51.2 | 2.6 | 7.48 | -0.49 | 10.0 | 58.0 |
| qBRCH8.1 | 2011 | 7.5 | 0-2.9 | 2.6 | 7.85 | 0.50 | 0.2 | 3.0 |
